# Supplementary material for: From sequence to enzyme mechanism using multi-label machine learning
Source: BMC Bioinformatics. 2014 May 19;15:150. doi: 10.1186/1471-2105-15-150 (PMC4229970; doi:10.1186/1471-2105-15-150)
Supplement: Additional file 2 — Java code of ml2db. Additional file ml2db_code.tar.gz contains the Java source code to run the multi-label machine learning experiments and save the results to database. The code’s Javadoc is included. [file 1471-2105-15-150-S2.zip › additional file 2/ml2db/utils/doc/cern/colt/class-use/PersistentObject.html]

Uses of Class cern.colt.PersistentObject


JavaScript is disabled on your browser.


- Overview
- Package
- Class
- Use
- Tree
- Deprecated
- Index
- Help

- Prev
- Next

- Frames
- No Frames

- All Classes

## Uses of Class cern.colt.PersistentObject

- Packages that use PersistentObject

  | Package | Description |
  | --- | --- |
  |  |  |
  | --- | --- |
  | cern.jet.random |  |
  | cern.jet.random.engine |  |
  | edu.cornell.lassp.houle.RngPack |  |
  | uk.ac.standrews.utils.main.stats |  |
- - ### Uses of PersistentObject in cern.jet.random

    Subclasses of PersistentObject in cern.jet.random

    | Modifier and Type | Class and Description |
    | --- | --- |
    |  |  |
    | --- | --- |
    | `class` | `AbstractContinousDistribution` Abstract base class for all continous distributions. |
    | `class` | `AbstractDistribution` Abstract base class for all random distributions. |
    | `class` | `Pareto` Pareto Distribution. |
  - ### Uses of PersistentObject in cern.jet.random.engine

    Subclasses of PersistentObject in cern.jet.random.engine

    | Modifier and Type | Class and Description |
    | --- | --- |
    |  |  |
    | --- | --- |
    | `class` | `MersenneTwister` MersenneTwister (MT19937) is one of the strongest uniform pseudo-random number generators known so far; at the same time it is quick. |
    | `class` | `RandomEngine` Abstract base class for uniform pseudo-random number generating engines. |
  - ### Uses of PersistentObject in edu.cornell.lassp.houle.RngPack

    Subclasses of PersistentObject in edu.cornell.lassp.houle.RngPack

    | Modifier and Type | Class and Description |
    | --- | --- |
    |  |  |
    | --- | --- |
    | `class` | `RandomElement` RandomElement is an abstract class that encapsulates uniform random number generators. |
    | `class` | `RandomJava` RandomJava is a class wrapper for the `Math.random()` generator that comes with Java. |
    | `class` | `RandomSeedable` `RandomSeedable` is an abstract class that extends the `RandomElement` class to include the ability to automatically generate a valid `long` seed from the clock. |
    | `class` | `RandomShuffle` RandomShuffle uses one random number generator to shuffle the numbers produced by another to obliterate sequential correlations. |
    | `class` | `Ranecu` Ranecu is an advanced multiplicative linear congruential random number generator with a period of aproximately 10^18^. |
    | `class` | `Ranlux` RANLUX is an advanced pseudo-random number generator based on the RCARRY algorithm proposed in 1991 by Marsaglia and Zaman. |
    | `class` | `Ranmar` RANMAR is a lagged Fibonacci generator proposed by Marsaglia and Zaman and is a good research grade generator. |
  - ### Uses of PersistentObject in uk.ac.standrews.utils.main.stats

    Subclasses of PersistentObject in uk.ac.standrews.utils.main.stats

    | Modifier and Type | Class and Description |
    | --- | --- |
    |  |  |
    | --- | --- |
    | `class` | `PseudoTruncatedPareto` A truncated pareto distribution which stores a big lists of power-law extracted random numbers. |
    | `class` | `TruncatedPareto` A utility class to extract from a truncated Pareto (power-law, long-tail) distribution. |

- Overview
- Package
- Class
- Use
- Tree
- Deprecated
- Index
- Help

- Prev
- Next

- Frames
- No Frames

- All Classes
